# Detecting differentially methylated loci for multiple treatments based on high-throughput methylation data

**DOI:** 10.1186/1471-2105-15-142

**Published:** 2014-05-15

**Authors:** Zhongxue Chen, Hanwen Huang, Qingzhong Liu

**Affiliations:** 1 Department of Epidemiology and Biostatistics, School of Public Health, Indiana University Bloomington, 1025 E. 7th street, PH C104, Bloomington, IN 47405, USA; 2 Department of Epidemiology and Biostatistics, University of Georgia, Athens, GA 30602, USA; 3 Department of Computer Science, Sam Houston State University, Huntsville, TX 77341, USA

**Keywords:** Cuzick test, Nonparametric test, Trend test

## Abstract

**Background:**

Because of its important effects, as an epigenetic factor, on gene expression and disease development, DNA methylation has drawn much attention from researchers. Detecting differentially methylated loci is an important but challenging step in studying the regulatory roles of DNA methylation in a broad range of biological processes and diseases. Several statistical approaches have been proposed to detect significant methylated loci; however, most of them were designed specifically for case-control studies.

**Results:**

Noticing that the age is associated with methylation level and the methylation data are not normally distributed, in this paper, we propose a nonparametric method to detect differentially methylated loci under multiple conditions with trend for Illumina Array Methylation data. The nonparametric method, Cuzick test is used to detect the differences among treatment groups with trend for each age group; then an overall p-value is calculated based on the method of combining those independent p-values each from one age group.

**Conclusions:**

We compare the new approach with other methods using simulated and real data. Our study shows that the proposed method outperforms other methods considered in this paper in term of power: it detected more biological meaningful differentially methylated loci than others.

## Background

DNA methylation is an epigenetic mark that has important effects on transcriptional regulation, chromosomal stability, genomic imprinting, and X-inactivation, [1,2]. In addition, it is associated with many human diseases, including various types of cancer [3-10].

Due to the recent advances of BeadArray technology, high-throughput genome-wide methylation data can be routinely generated by Infinium Methylation Assays. This provides good opportunities for researchers to simultaneously study hundreds of thousands of DNA methylation loci. However, it also requires sophisticated and advanced statistical methods to analyze this kind of data.

The raw data generated from BeadArray are fluorescent intensities for each locus; they need appropriate preprocesses, such as background correction and normalization. Then a summarized β-value is generated from about 30 replicates in the same array as follows: $\beta =\frac{\max\left(M,0\right)}{\max\left(U,0\right)+100}$, where M is the average signal from a methylated allele while U is that from an unmethylated allele. Obviously, the β-values are continuous numbers between 0 and 1, with 0 stands for totally unmethylated and 1 for completely methylated.

Due to the non-normality of the β-value [11-13], those commonly used statistical methods, such as t-test for case control designs, ANOVA for multiple conditions, or linear regression with age as a predictor, usually have low power to detect differentially methylated loci [13,14]. Some statistical approaches with or without adjusting the age-effect, which has been found highly associated with DNA methylation [15,16], have been proposed to detect differentially methylated loci for case-control designs [11-13]. However, very little work has been done for the situation where there are three or more groups (e.g., conditions, or treatments). In a previous study, we compared some statistical tests with age-effect adjustment for DNA methylation data with three treatments, and found that the method based on the nonparametric Kruskal-Wallis (KW) test is usually more powerful than other methods, such as ANOVA and regression method [14]. However, if there is a trend associated with treatments or conditions, KW based test is no longer the optimal method since it ignores this information. In this case, a more powerful statistical approach is desirable.

In this paper, we propose a new statistical approach to detecting differentially methylated loci for methylation data with multiple conditions with trend. In this method, we also adjust the age-effect in a similar way that we used before. More specifically, we first group subjects into several categories based on their age; we then apply a nonparametric trend test and get a one-sided p-value for each age category. An overall p-value is then obtained through combining those individual p-values. The performance of the new approach is assessed through comparing it with other methods using simulated data and a real methylation data set with three treatments. The R code for the new method is provided (please see the Additional file 1: R code).

## Methods

### Existing methods

In a recent paper, we have proposed several methods based on combining independent p-values to adjust the effect of age for genome-wide methylation data with multiple conditions [14]. Since those commonly used methods, such as regression models with age as continuous or categorical covariate, have poor performances [12], we compare the proposed approach with the following ones, which have the best performances among current methods based on our previous study [14].

### Combined ANOVA test

We assume there are *K* conditions (treatments) and *G* age groups. For each age group *g (g = 1,2,…,G)*, we apply an ANOVA test and obtain a p-value $p_{g}^{A}\mathrm{NOVA}.$ We know that under the null hypothesis that this locus is not differentially methylated among K conditions in any age group, $-2\log\left(\prod_{g=1}^{G}p_{g}^{\mathrm{ANOVA}}\right)$ has a chi-square distribution with 2G degrees of freedom (df) since these G p-values are independent. Therefore, the overall p-value can be obtained through combining p-values by Fisher test [17]:

(1) $$p_{\mathrm{ANOVA}}=\chi_{\mathrm{df}=2G}^{2}\left(\chi^{2}>-2\sum_{g=1}^{G}\log\left(p_{g}^{\mathrm{ANOVA}}\right)\right)$$

### Combined KW test

Similarly, we replace ANOVA by the nonparametric Kruskal-Wallis test for each age group and obtain an overall p-value with $p_{g}^{\mathrm{ANOVA}}$ being replaced by the p-value $p_{g}^{\mathrm{KW}}$ from KW test:

(2.) $$p_{\mathrm{KW}}=\chi_{\mathrm{df}=2G}^{2}\left(\chi^{2}>-2\sum_{g=1}^{G}\log\left(p_{g}^{\mathrm{KW}}\right)\right)$$

### Methods for combining p-values

Besides the Fisher method mentioned above, we can also use Z-test to combine p-values from independent tests [18-20]. We calculated the weighted Z statistic using individual p-values from each age group: $Z=\sum_{g=1}^{G}n_{g}\Phi^{-1}\left(1-p_{g}\right)/\sum_{g=1}^{G}n_{g}^{2}$, where *n*_*g*_ is the total sample size in age group *g* and Φ is the cumulative distribution function (CDF) of the standard normal distribution. It can be shown that under the null hypothesis this statistic has the standard normal distribution. The overall p-value is then calculated as 1- Φ(Z) by the one-sided z-test.

### The proposed method

If there is a monotonic trend of the outcome (i.e., β-value here) over the treatments, we can use the more powerful one-sided Cuzick test [21] to obtain a p-value for each age group g (g = 1,2,…,G). The Cuzick test statistic for age group g is calculated as:

(3) $$C_{g}=\frac{\sum_{i=1}^{N_{g}}r_{\mathrm{gi}}s_{\mathrm{gi}}-N_{g}\left(N_{g}+1\right)\sum_{i=1}^{K}s_{\mathrm{gi}}p_{\mathrm{gi}}/2}{\sqrt{\frac{1}{12}{N_{g}}^{2}\left(N_{g}+1\right)\left(\sum_{i=1}^{K}s_{\mathrm{gi}}^{2}p_{\mathrm{gi}}-{\left(\sum_{i=1}^{K}s_{\mathrm{gi}}p_{\mathrm{gi}}\right)}^{2}\right)}}$$

where N_g_ is the total number of subjects in age group g, *r*_*gi*_ is the rank of the i^th^ of the N_g_ subjects, *s*_*gk*_ is the score of the k^th^ (*k* = 1, 2, …, *K*) treatment, K is the number of treatments, $p_{\mathrm{gk}}=\frac{n_{\mathrm{gk}}}{N_{g}}$, and *n*_*gk*_ is the number of subjects in the k^th^ treatment within the g^th^ age group. For the k^th^ treatment, we assign a score *s*_*gk*_ to each of the *n*_*gk*_ subjects. In this paper, we set *s*_*gk*_ = *k* (*k* = 1, 2, …, *K*), that is, we use scores 1,2,…,K.

It can be shown that under the null hypothesis, the statistic C_g_ (g = 1,2,…,G ) in (3) has an asymptotic standard normal distribution [21]. If there is an increasing trend over the K treatments, we should use the one-sided p-value, *p*_*r*,*g*_ = *Prob*(*Z* > *c*_*g*_) = 1 - Φ(*c*_*g*_). On the other hand if there is a decreasing trend over the K treatments, we should use the other one-sided p-value, *p*_*l*,*g*_ = *Prob*(*Z* < *c*_*g*_) = Φ(*c*_*g*_). The statistic $\;W_{1}=-2\log\left(\prod_{g=1}^{G}p_{l,g}\right)$ has an asymptotic chi-square distribution with 2G df under the null hypothesis according to Fisher [17]. Similarly, under the null hypothesis the statistic $W_{2}=-2\log\left(\prod_{g=1}^{G}\left(1-p_{l,g}\right)\right)$ also has an asymptotic chi-square distribution with 2G df.

If we know the direction of the trend (increasing or decreasing), we should use either  *W*_1_ or  *W*_2_ to calculate the overall p-value. However, if the trend direction is unknown, which is usually the case in practice, we will use the maximum of the two statistics:

(4) $$W=\max\;\left(W_{1},W_{2}\right)$$

Since *W*_1_ and  *W*_2_ are correlated, the null distribution of *W* is not easy to obtain. However, we have the following nice result [22-27].

**Theorem 1** Under the null hypothesis, the survival function of *W* in (4) is asymptotically bounded by

(5) $$2\gamma -\gamma^{2}\leq \mathrm{Pr}\left(W>w\right)\leq 2\gamma ,$$

where $\gamma =1-\chi_{2G}^{2}\left(w\right),$ and $\chi_{2G}^{2}\left(.\right)$ is the cumulative distribution function of the chi-square distribution with 2G df.

The above theorem can be proved using the concept of associated variables due to Esary, Proschan and Walkup [28] and Theorem 2 of Owen [29]. From theorem 1, the overall p-value can be estimated by the upper bound 2*γ*. It is easily seen that when the true p-value of W is small, the difference between the true and the estimated p-values is negligible.

We can also estimate the overall p-value by the weighted Z-test:

(6) $$p_{Z}=1-\Phi \left(\left|\sum_{g=1}^{G}w_{g}c_{g}\right|\right)$$

where the weight $w_{g}=\frac{n_{g}}{N}$, and *n*_*g*_ (g = 1,2,…,G) is the number of total subjects of the K treatments within the g^th^ age group. The validity of (6) is easily seen: under the null hypothesis *c*_*g*_ and therefore *w*_*g*_*c*_*g*_ has an asymptotic standard normal distribution; a two-sided p-value then can be obtained through (6).

### Simulation settings

To assess the performance of the proposed method, we use simulated data to compare the proposed test with current methods in terms of controlling type I error rate and power. We assume there are three different treatments (i..e, K = 3) and six age groups (i.e., G = 6). For each treatment we assume the β-value has the same following distributions over the six age groups: (i) uniform U(a,b) where 0 ≤ a < b ≤ 1, (ii) truncated normal TN (*μ*, *σ*^2^, 0, 1) (or simply TN (*μ*, *σ*^2^), and (iii) Beta distribution Beta (c,d) with various parameters. We consider relatively small sample sizes in our simulation study. To reflect practical situations, we either choose 20 samples for each of the three treatments (sample size = (20, 20, 20)), or set the sample sizes as 15, 20, and 25 (sample size = (15, 20, 25)), respectively, for the three treatments. Since the proposed test is designed to detect differentially methylated loci when there is a monotonic trend over the treatments, we simulate β-values with increasing or decreasing mean values over the three treatments for the alternative hypotheses. For example, in simulation, we first generate 20 β-values (sample size = (20, 20, 20)) from three uniform distributions (denoted by a = (0,0,0.25), b = (1,1,1)), U(0,1), U(0,1), and U(0.25, 1) for each of the three treatment groups. The significance level is set to be 0.05 in simulation study. The type I error rate and power are estimated by the proportions of rejection with 10^4^ replicates.

### A real data set

The real methylation data set of the United Kingdom Ovarian Cancer Population Study (UKOPS) [16], which is one of the largest available Illumina methylation data sets, will be used for real data application. This data set originally includes 274 healthy controls, 131 pre-treatment cases, and 135 post treatment cases. If the DNA methylation of a locus is positively associated with the disease, we would expect that the methylation rates are increasing from control to post-treatment then to pre-treatment. On the other hand, if the association is negative, there would be a decreasing trend over the three conditions: control, post-treatment, and pre-treatment. In either of the two situations, we can use the proposed test.

The above mentioned methylation data were generated by the Illumina Infinium Human Methylation27 BeadChip and can be downloaded from the NCBI Gene Expression Omnibus (http://www.ncbi.nlm.nih.gov/geo) with the accession number GSE19711. For this data set, there are 27578 loci. After a data quality control process, we removed 60 subjects with BS values less than 4000 or the coverage rates less than 95%. All of the subjects are separated into 6 age groups (50-55, 55-60, 60-65, 65-70, 70-75, and 75 and over). Table 1 lists the resulting numbers of subjects in each age by treatment group. For each locus, we apply the proposed test and other methods.

**Table 1:** Number of samples in age by treatment group used in the paper after data quality control step

**Age group**	**Control**	**Pre-treat**	**Post-treat**	**Total**
50_55	14	15	16	45
55_60	61	17	25	103
60_65	64	17	22	103
65_70	35	17	21	73
70_75	63	24	22	109
75_over	20	18	9	47
Total	257	108	115	480

## Results

### Simulation results

For the new method and the combined ANOVA and KW tests, we only report the results using Fisher method to combine independent p-values, as the results using Z-test are very similar. Table 2 reports the empirical type I error rates for the proposed method, the combined ANOVA test and the combined KW test, from the simulation study. It is clearly shown that even if the sample size is relatively small and the underlying distribution is not normal, all the methods, including the ANOVA based test, control type I error rate quite well.

**Table 2:** **Empirical size for each method at significance level 0.05 with 10**^**4 **^**replicates from the simulation study**

**Simulation setting (3 treatments)**			**Combined ANOVA**	**Combined K-W**	**New**
**Distribution**	**Sample size**	**Parameters**			
Uniform U(a,b)	(20,20,20)	a = (0,0,0), b = (1,1,1)	0.051	0.045	0.047
		a = (0,0,0), b = (0.5,0.5,0.5)	0.051	0.045	0.045
		a = (0.5,0.5,0.5), b = (1,1,1)	0.055	0.046	0.047
	(15,20,25)	a = (0,0,0), b = (1,1,1)	0.052	0.043	0.046
		a = (0,0,0), b = (0.5,0.5,0.5)	0.052	0.044	0.050
		a = (0.5,0.5,0.5), b = (1,1,1)	0.049	0.040	0.043
Truncated Normal TN (μ ,σ^2^)	(20,20,20)	μ = (0.5,0.5,0.5), σ = (1,1,1)/5	0.050	0.043	0.048
		μ = (0.5,0.5,0.5), σ = (1,2,3)/5	0.058	0.050	0.045
		μ = (0.2,0.2,0.2), σ = (1,1,1)/5	0.049	0.050	0.043
		μ = (0.8, 0.8, 0.8), σ = (1,1,1)/5	0.046	0.043	0.048
	(15,20,25)	μ = (0.5,0.5,0.5), σ = (1,1,1)/5	0.050	0.046	0.045
		μ = (0.5,0.5,0.5), σ = (1,1.2,1.3)/5	0.053	0.041	0.033
		μ = (0.2,0.2,0.2), σ = (1,1,1)/5	0.050	0.046	0.051
		μ = (0.8, 0.8, 0.8), σ = (1,1,1)/5	0.049	0.044	0.048
Beta (c,d)	(20,20,20)	c = (1,1,1), d = (1,1,1)	0.050	0.044	0.049
		c = (1,1,1), d = (5,5,5)	0.046	0.045	0.043
		c = (5,5,5), d = (1,1,1)	0.048	0.044	0.045
		c = (5,5,5), d = (5,5,5)	0.049	0.041	0.044
	(15,20,25)	c = (1,1,1), d = (1,1,1)	0.049	0.044	0.046
		c = (1,1,1), d = (5,5,5)	0.045	0.042	0.047
		c = (5,5,5), d = (1,1,1)	0.049	0.049	0.048
		c = (5,5,5), d = (5,5,5)	0.052	0.044	0.052

Table 3 lists the empirical power values for the three methods under various situations. As expected, the proposed test always has higher power values than those of the combined ANOVA and KW tests. This demonstrates that the proposed test which uses the trend information can improve the detecting power. It should point out that in the simulation study, we assign scores 1, 2, and 3 to the three treatments. However, the effect sizes between treatments 1 and 2 and that between treatments 2 and 3 are not set to be 1 to 2, respectively, which makes the scores (1,2,3) optimal; therefore, the proposed test have the best power. In words, we don’t use the optimal scores for the Cuzick test to reflect the real situations when the optimal scores are unknown. This can be seen from the powers of the new test with different scores (e.g., (1,1,2), and (1,3,2)) in the last two columns of Table 3. For many situations considered in Table 3, the scores (1,1,2) are closer to the optimal scores, which are determined by the effect sizes of treatments 2 vs. 1, and treatments 3 vs. 1, than the default ones, (1,2,3); therefore, it is not surprising that the new test with scores (1,1,2) has larger power values than those from the one with scores (1,2,3). However, for most of the situations, the scores (1,3,2) do not use the trend correctly and hence has lower power compared with the other two.

**Table 3:** **Empirical power for each method at significance level 0.05 with 10**^**4 **^**replicates from the simulation study**

**Simulation setting (3 treatments)**			**Combined ANOVA**	**Combined K-W**	**New**^**1**^	**New**^**2**^	**New**^**3**^
**Distribution**	**Sample size**	**Parameters**					
Uniform U(a,b)	(20,20,20)	a = (0,0,0.25), b = (1,1,1)	0.699	0.607	0.877	0.962	0.069
		a = (0,0.1,0.1), b = (0.5,0.5,0.5)	0.450	0.339	0.724	0.830	0.726
		a = (0.6,0.6,0.5), b = (1,1,1)	0.460	0.338	0.695	0.821	0.027
	(15,20,25)	a = (0,0,0.25), b = (1,1,1)	0.809	0.692	0.926	0.980	0.957
		a = (0,0.1,0.1), b = (0.5,0.5,0.5)	0.433	0.319	0.618	0.758	0.218
		a = (0.6,0.6,0.5), b = (1,1,1)	0.482	0.380	0.754	0.854	0.860
Truncated Normal TN (*μ*, *σ*^2^)	(20,20,20)	*μ* = (0.1,0.1,0.2), *σ* = (1,1,1)/5	0.451	0.394	0.743	0.862	0.052
		*μ* = (0.1,0.1,0.2), *σ* = (1,1.2,1.3)/5	0.773	0.642	0.962	0.954	0.200
		*μ* = (0.5,0.5,0.4), *σ* = (1,1,1)/5	0.691	0.656	0.918	0.976	0.054
		*μ* = (0.5,0.5,0.4), *σ* = (1,1.2,1.3)/5	0.402	0.374	0.696	0.820	0.032
	(15,20,25)	*μ* = (0.1,0.1,0.2), *σ* = (1,1,1)/5	0.464	0.428	0.786	0.886	0.948
		*μ* = (0.1,0.1,0.2), *σ* = (1,1.2,1.3)/5	0.735	0.643	0.959	0.952	0.713
		*μ* = (0.5,0.5,0.4), *σ* = (1,1,1)/5	0.775	0.738	0.949	0.981	0.827
		*μ* = (0.5,0.5,0.4), *σ* = (1,1.2,1.3)/5	0.382	0.364	0.756	0.838	0.852
Beta (c,d)	(20,20,20)	c = (1,1,1), d = (30,40,50)	0.596	0.442	0.889	0.723	0.432
		c = (1,1.2,1.5), d = (40,40,40)	0.490	0.609	0.962	0.920	0.329
		c = (30,40,50), d = (1,1,1)	0.578	0.450	0.899	0.745	0.420
		c = (40,40,40), d = (1,1.2,1.5)	0.488	0.620	0.972	0.924	0.369
	(15,20,25)	c = (1,1,1), d = (30,40,50)	0.608	0.405	0.861	0.727	0.998
		c = (1,1.2,1.5), d = (40,40,40)	0.426	0.602	0.952	0.912	0.559
		c = (30,40,50), d = (1,1,1)	0.618	0.409	0.888	0.752	0.458
		c = (40,40,40), d = (1,1.2,1.5)	0.450	0.606	0.958	0.919	0.995

Note: ^1^the prosed test with scores (1,2,3), ^2^the prosed test with scores (1,1,2), ^3^the prosed test with scores (1,3,2).

### Results from the real data application

The proposed test and the combined ANOVA and KW tests are applied to the real data mentioned above. Due to the multiple comparison issue and the correlation among loci, it is desirable but difficult to obtain a meaningful cutoff p-value to determine differentially methylated loci. We therefore report the numbers of loci with p-values less than a given cutoff value from each method. We choose different cutoff values: 10^-3^, 10^-4^, 10^-5^, 10^-6^, 10^-7^, and 10^-8^. The results are reported in Table 4. For each of the given cutoff p-values, the proposed test always detects more loci than the other methods. In addition, most of the loci detected by the combined ANOVA and KW tests were also detected by the proposed test. For example, when the cutoff p-value is 10^-5^, the combined ANOVA test , the combined KW test, and the proposed test detected 479, 551, and 1283 loci, respectively, when Fisher method was used to combine p-values. Out of the 479 loci detected by the combined ANOVA test, 471 were also detected by the new test; out of the 551 loci detected by the combined KW test, only 7 were not detected by the proposed test.

**Table 4:** Number of significant differentially methylated loci detected by each method for each given cutoff p-value

**Method**	**1e-3**		**1e-4**		**1e-5**		**1e-6**		**1e-7**		**1e-8**	
	**F**	**Z**	**F**	**Z**	**F**	**Z**	**F**	**Z**	**F**	**Z**	**F**	**Z**
T1 (Combined ANOVA)	981	1079	655	690	479	499	350	375	257	275	189	208
T2 (Combined KW)	1359	1340	823	859	551	590	381	401	261	277	172	185
T3 (New)	2915	3117	1855	1951	1283	1310	905	929	674	686	513	521
T1 and T2	926	980	615	656	442	474	306	338	221	235	152	167
T1 and T3	931	1018	639	670	471	491	346	367	252	269	187	206
T2 and T3	1294	1279	806	832	544	577	377	396	259	276	170	184
T1, T2, and T3	895	954	605	642	437	468	303	336	220	234	151	166

This indicates that the proposed test is more powerful than other methods that are compared in this study. It is noticeable that the methods of combining independent p-values (i.e., Fisher test and Z-test) have similar performance here, although the Z-test usually gives a few more significant loci.

## Discussion

**Figure 1:**
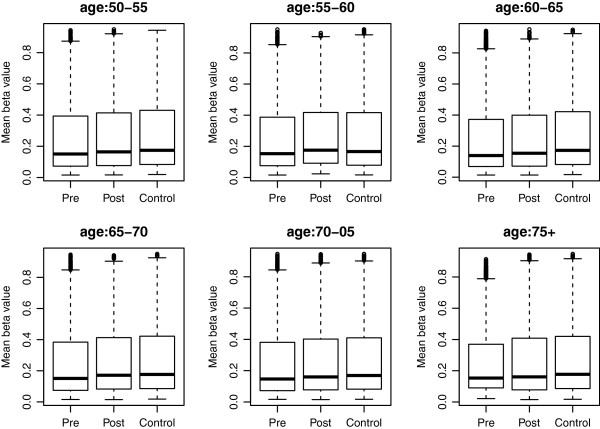
**The mean β-value of loci with p-value less than 10**^**-3 **^**from the proposed test over the three treatment groups by the age group.** For each age group, there is a trend among the three treatments: pre-treatment has smaller β-value than the post-treatment group, which in turn has smaller β-value than the control group.

We proposed a new statistical approach based on combing p-values and the Cuzick test, which is a nonparametric one-sided test. Through simulation study and real data application, we show that if there exists a monotonic (not necessarily linear) trend over the treatments, the proposed test is more powerful than other methods. Figure 1 plots the mean β-value of each of the three treatments over the six age groups for loci with p-values less than 10^-3^ from the proposed test. From Figure 1, we can see there is a decreasing trend among the three treatments (i.e., for the β-value, pre-treatment < post-treatment < control) for all of the six age groups; while from for those loci with large p-values, such trend does not exist for any of the six age groups (see Additional file 2: Figure S1).

Although many methods can detect those loci which are strongly differentially methylated among different treatments, it is important to detect loci having small effects as they are biological meaningful and provide useful information for set-based analyses, such as gene, gene-set, and pathway analyses which use those detected differentially methylated loci as input [30].

To use the Cuzick test, we need to assign a score for each of the treatment. Here we assign 1, 2, and 3 to the control, post-treatment, and pre-treatment, respectively. In practice, if we have the information of the effects for each treatment, we can use this information to assign scores. For example, for the K-1 treatments 2, 3, …, K, if the effect sizes are m_2_, …, m_K_ compared to treatment 1, we can assign scores 0, m_2_, …, m_K_ to those treatments for the proposed test. However, if we only know that there is a monotonic trend, we can choose 1, 2, …, K (equivalent to 0, 1, …, K-1) as the scores. Although, the performance of the proposed test can be improved by assigning optimal scores, which are determined by the true effects, to the treatments; in general, it is impractical to obtain the optimal scores. In addition, the optimal scores for each locus may not be the same across age groups (see Figure 1).

Like other large scale data, such as microarray data and genome-wide association study data, the multiple comparison is an important but challenging issue. Although some procedures have been proposed to control either family-wise error rate or false discovery rate, it remains an open topic in this area. One possible direction is to use the so-called “effective number” estimated from correlations among the loci [31].

## Conclusions

We propose a new statistical approach to detecting methylated loci for high-throughput methylation data with multiple groups. This approach is based on the nonparametric Cuzick test, which is robust and powerful if there exists a trend over groups. Through simulated and real data, we show that the proposed test outperforms existing methods.

## Competing interests

The authors declare that they have no competing interests.

## Authors’ contributions

ZC, HH, and QL were jointly responsible for the development of the algorithm and the writing of the manuscript. All authors read and approved the final manuscript.

## Supplementary Material

Additional file 1 R code. Click here for file

Additional file 2: Figure S1 The mean β-value of loci with p-value greater than 10^-3^ from the proposed test over the three treatment groups by the age group. For each age group, there is no obvious trend over the three treatments for the β-value. Click here for file
