# Supplementary material for: Detecting differentially methylated loci for multiple treatments based on high-throughput methylation data
Source: BMC Bioinformatics. 2014 May 15;15:142. doi: 10.1186/1471-2105-15-142 (PMC4026834; doi:10.1186/1471-2105-15-142)
Supplement: Additional file 2: Figure S1 — The mean β-value of loci with p-value greater than 10-3 from the proposed test over the three treatment groups by the age group. For each age group, there is no obvious trend over the three treatments for the β-value. [file 1471-2105-15-142-S2.pdf]

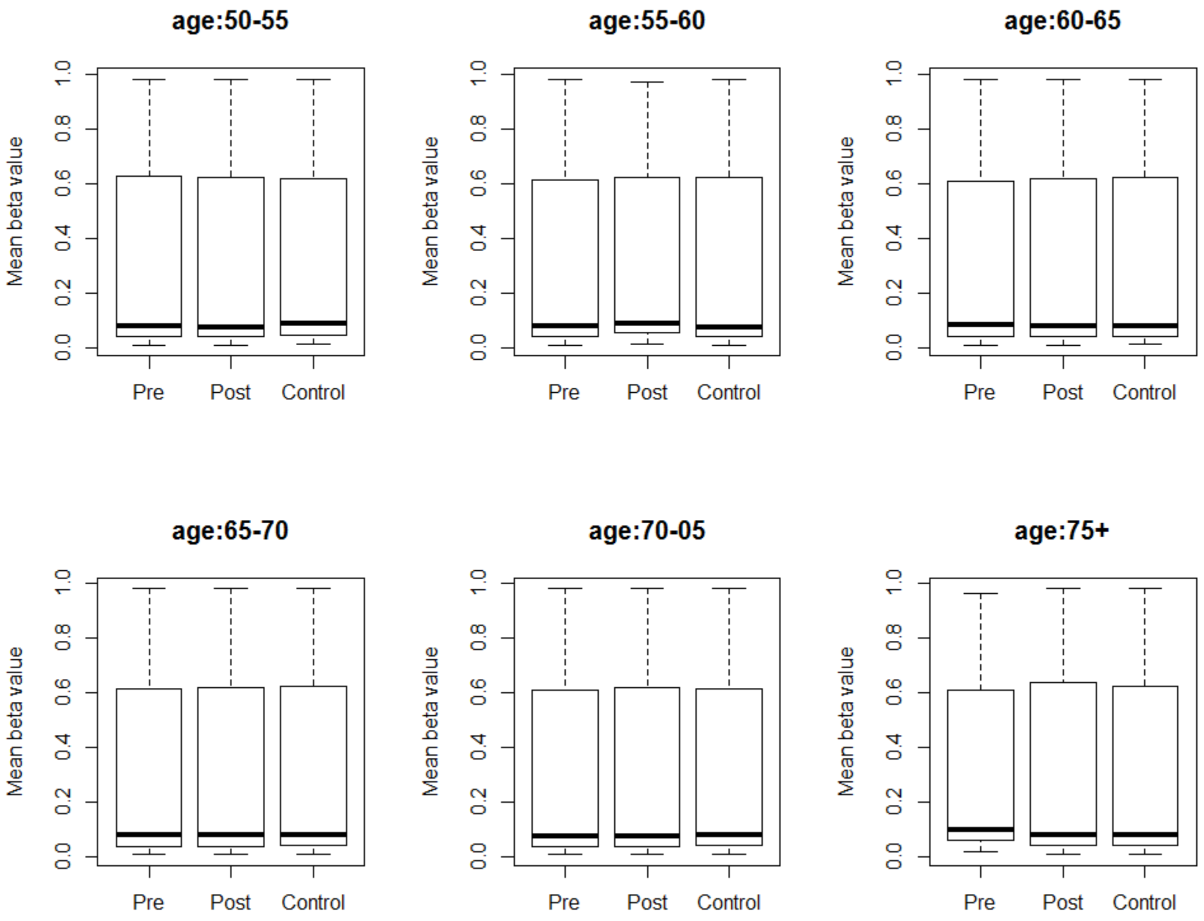

Supplementary Figure 1. The mean β-value of loci with p-value greater than 10^{-3} from the proposed test over the three treatment groups by the age group. For each age group, there is no obvious trend over the three treatments for the β-value.
